# Screening for SARS-CoV-2 Infection Within a Psychiatric Hospital and Considerations for Limiting Transmission Within Residential Psychiatric Facilities — Wyoming, 2020

**DOI:** 10.15585/mmwr.mm6926a4

**Published:** 2020-07-03

**Authors:** Anna W. Callaghan, Anna N. Chard, Patricia Arnold, Cody Loveland, Noah Hull, Mona Saraiya, Sharon Saydah, Wendy Dumont, Laura G. Frakes, Daniel Johnson, ReaAnna Peltier, Clayton Van Houten, A. Angelica Trujillo, Jazmyn Moore, Dale A. Rose, Margaret A. Honein, David Carrington, Alexia Harrist, Susan L. Hills

**Affiliations:** ^1^CDC Wyoming COVID-19 Response Field Team; ^2^Epidemic Intelligence Service, CDC; ^3^Wyoming State Hospital, Evanston, Wyoming; ^4^Wyoming Department of Health; ^5^CDC COVID-19 Response Team.

In the United States, approximately 180,000 patients receive mental health services each day at approximately 4,000 inpatient and residential psychiatric facilities (*1*). SARS-CoV-2, the virus that causes coronavirus disease 2019 (COVID-19), can spread rapidly within congregate residential settings (*2*–*4*), including psychiatric facilities. On April 13, 2020, two patients were transferred to Wyoming’s state psychiatric hospital from a private psychiatric hospital that had confirmed COVID-19 cases among its residents and staff members (*5*). Although both patients were asymptomatic at the time of transfer and one had a negative test result for SARS-CoV-2 at the originating facility, they were both isolated and received testing upon arrival at the state facility. On April 16, 2020, the test results indicated that both patients had SARS-CoV-2 infection. In response, the state hospital implemented expanded COVID-19 infection prevention and control (IPC) procedures (e.g., enhanced screening, testing, and management of new patient admissions) and adapted some standard IPC measures to facilitate implementation within the psychiatric patient population (e.g., use of modified face coverings). To assess the likely effectiveness of these procedures and determine SARS-CoV-2 infection prevalence among patients and health care personnel (HCP) (*6*) at the state hospital, a point prevalence survey was conducted. On May 1, 2020, 18 days after the patients’ arrival, 46 (61%) of 76 patients and 171 (61%) of 282 HCP had nasopharyngeal swabs collected and tested for SARS-CoV-2 RNA by reverse transcription–polymerase chain reaction. All patients and HCP who received testing had negative test results, suggesting that the hospital’s expanded IPC strategies might have been effective in preventing the introduction and spread of SARS-CoV-2 infection within the facility. In congregate residential settings, prompt identification of COVID-19 cases and application of strong IPC procedures are critical to ensuring the protection of other patients and staff members. Although standard guidance exists for other congregate facilities (*7*) and for HCP in general (*8*), modifications and nonstandard solutions might be needed to account for the specific needs of psychiatric facilities, their patients, and staff members.

Wyoming’s state psychiatric hospital is a complex residential facility comprising three very different units: Adult Psychiatric Services, Medical Geriatric Psychiatric Services, and Criminal Justice Services. Each unit presents its own multifaceted challenges in terms of patient populations, the level of care required, and the risks to HCP posed by patient behaviors, which are not typically encountered in other residential settings. Patients at the state hospital are all aged ≥19 years, and admissions come from other health care, group residential, and correctional facilities within the state. Approximately 300 staff members are employed at the hospital, mostly HCP who provide varying levels of patient care. The hospital currently has a 104-bed capacity, with approximately 65% of beds in double occupancy rooms.

The state hospital had no known COVID-19 cases among patients or staff members before the transfer of two patients from a private psychiatric hospital on April 13, 2020. In late March, the state hospital had started some testing of new admissions and patients with COVID-19–like symptoms, and staff members had been advised to seek testing through their primary care providers if they had symptoms suggestive of COVID-19, but no cases had been identified. In early April, the originating private hospital had performed SARS-CoV-2 testing for one of the transferred patients when planning for transfer to an alternative facility and received a negative result on April 3, 2020; no testing was performed for the second patient before transfer to the state hospital. Because of the reported COVID-19 outbreak at the private facility (*5*), both patients were tested at the time of admission to the state hospital. While awaiting test results, both patients were isolated in separate rooms as a precautionary measure, and any staff members with exposure to the two patients during transport or admission while not wearing all recommended personal protective equipment (PPE) (*8*) were asked to self-quarantine.

When both patients received positive laboratory test results, the state hospital immediately implemented enhanced measures to prevent further transmission, including 1) the screening and testing of all new patients by a dedicated admissions team, 2) immediate isolation of new patients in separate rooms until receipt of test results, and 3) isolation and management of new patients with positive SARS-CoV-2 test results in a separate ward (supported by eight dedicated nurses who provided clinical care and housekeeping services) for 2 weeks or until receipt of two negative SARS-CoV-2 results from nasopharyngeal swab specimens collected 24 hours apart.* These same procedures were also to be followed if symptoms were identified in any patients already at the state hospital. In addition, standard IPC procedures already in place were reinforced, based on long-term care facility guidelines (*7*), including 1) universal cloth face coverings for compliant patients and face masks for HCP at all times within the facility, 2) frequent disinfection of spaces accessed by patients with COVID-19 and all communal spaces, 3) cancellation of group dining or increase of space between patients at dining tables, 4) reduction in the number of persons participating in group therapy sessions, 5) limitation of all nonessential visitors and services, and 6) daily symptom screening and temperature checks of all patients and staff members (*7*). Some standard IPC measures were modified to facilitate implementation in the psychiatric patient population, such as adapting face coverings for patients to avoid elastic and metal components that could be used for self-harm or violent purposes (e.g., socks, a preapproved item, were modified for use as face coverings).

On May 1, 2020, the state hospital, with support from the Wyoming Department of Health and CDC, conducted a point prevalence survey to determine the prevalence of SARS-CoV-2 infection among patients and HCP and to assess the effectiveness of the newly implemented enhanced patient admission, isolation, and IPC procedures. All state psychiatric hospital patients and HCP (*6*) were invited to participate in the survey. Two-person survey teams were located in each of the hospital’s three units. Participants provided oral consent for survey participation; per hospital policy, hospital staff members obtained guardian consent before the survey for any patients with legal guardians. Survey team members administered a questionnaire to patients and HCP that elicited information about demographic characteristics, patient’s unit, symptoms, and HCP duties and work locations in the past 2 weeks. Survey team members also collected one nasopharyngeal swab specimen from each participant. Specimens were tested at the Wyoming State Public Health Laboratory using the CDC 2019 Novel Coronavirus (2019-nCoV) Real-Time Reverse Transcriptase (RT)–PCR Diagnostic Panel (*9*). This survey was conducted by a public health authority to provide timely situational awareness and priority setting during the COVID-19 pandemic, and as such, was considered nonresearch public health surveillance as outlined in 45 CFR 46.102(l)(2).^†^

Overall, 46 (61%) of 76 patients and 171 (61%) of 282 HCP participated in the survey and had nasopharyngeal swab specimens collected and tested (Table 1) (one clinical care staff member was excluded because their sample was received at the laboratory without a label). Included among the 76 patients were 21 (68%) of 31 in the Adult Psychiatric Services unit, 16 (76%) of 21 in the Medical Geriatric Psychiatric Services unit, and nine (38%) of 24 patients in the Criminal Justice Services unit. Included among the 171 HCP were 137 (58%) of 238 in clinical care, 14 (88%) of 16 in housekeeping, and 20 (74%) of 27 in transportation and security. Median length of patient stay was 150 days (interquartile range = 86–381 days). Among the 171 participating HCP, 151 (88%) reported providing direct care to the patients, eight (5%) reported working within another health care facility, and 98 (57%) reported working across multiple units at the state hospital within the previous 2 weeks. Responses to survey questions regarding COVID-19–like symptoms were inconsistent and incomplete because patients and staff members would often mention non-COVID-19–like symptoms or attribute symptoms to existing comorbidities; therefore, these responses were not included in the analysis. All patients and HCP had negative test results for SARS-CoV-2 infection.

**TABLE 1:** Characteristics of patients and health care personnel (HCP) who participated in the point prevalence survey at a state psychiatric hospital — Wyoming, May 1, 2020

Patient characteristic	Hospital service unit or role			
	Adult psychiatric	Medical geriatric psychiatric	Criminal justice	Total patients
No. participating/Total no.	21/31	16/21	9/24	46/76
Male, no. (%)	8 (38)	4 (25)	6 (67)	18 (39)
Median age, yrs (IQR)	48 (38–61)	62 (57–66)	42 (32–59)	57 (41–63)
Median length of admission, days (IQR)	107 (76–176)	320 (121–735)	150 (73–228)	150 (86–381)
**HCP characteristic**	**Clinical care**	**Housekeeping**	**Transport/Security**	**Total HCP**
No. participating/Total no.	137/238	14/16	20/27	171/282*
Male, no. (%)	37 (27)	0 (0)	13 (65)	50 (29)
Median age, yrs (IQR)	41 (32–54)	55 (43–57)	46 (34–53)	43 (32–55)
Provided direct patient care, no. (%)^†^	132 (96)	2 (14)	18 (90)	151 (88)
Worked at other health care facilities within previous 2 weeks, no. (%)	7 (5)	0 (0)	1 (5)	8 (5)
Worked on multiple units at the state hospital within previous 2 weeks, no. (%)	72 (53)	10 (71)	17 (85)	98 (57)

**Abbreviations**: HCP = health care personnel; IQR = interquartile range.

*One HCP staff member was excluded because the nasopharyngeal sample arrived at the testing laboratory without a label.

^†^ As reported by HCP; at times housekeeping, transportation, and security staff members might provide nonclinical direct patient care, such as assisting the patients to move around the facility or intervening if a patient becomes violent.

Based on observations and discussions at the state psychiatric hospital and review of reports from other facilities in Wyoming, various unique concerns were identified related to preventing and managing SARS-CoV-2 transmission in psychiatric facilities (Table 2). In the rapidly evolving early days of the COVID-19 pandemic, the psychiatric facilities in Wyoming were faced with the task of adapting standard IPC procedures to their specific settings, given the needs of their patient population, the specific risks for their staff members, and the limitations of their physical facilities. These concerns were tabulated and organized in terms of provider group and processes, and possible solutions were proposed. The issues faced ranged from the ability to cohort infected patients when it was also necessary to segregate patients by age, gender, and treatment needs, to the ability to continue essential mental health services when physical distancing or isolation had to be maintained.

**TABLE 2:** Infection prevention, control, and other considerations based on observations at psychiatric facilities during the COVID-19 pandemic — Wyoming, May 2020

Group/Process	Challenges to effective COVID-19 prevention and control	Possible solutions
**Patients**		
Admissions	Admissions from facilities at higher risk for SARS-CoV-2 transmission (e.g., homeless shelters, group homes, and correctional facilities)	Test newly admitted patients to identify any persons with asymptomatic infection and defer integration to regular wards until results are received. If result is positive, keep patient isolated; if result is negative, conduct routine symptom screening on regular ward
Screening	Uncooperative/violent behavior when patients are being screened for symptoms or tested for SARS-CoV-2 infection	Educate patients to raise awareness of the need for screening and testing, and to avoid misinformation and fear
Cohorting	Logistical challenge to segregate according to age, gender, treatment needs, and potential for violence in addition to cohorting based on COVID-19 case status	Implement rigorous measures to prevent transmission into and within the facility to avoid the need for patient cohorting in addition to the normal necessary segregation of patients. If transmission occurs, isolate patients in single rooms, or in rooms with other COVID-19 patients as segregation of patients allows, within quarantined areas to limit interaction
Social distancing	Psychiatric treatment often requires close interaction and cannot be canceled or delayed	Conduct smaller group sessions or one-on-one therapy, with 6-foot distancing, universal use of face coverings, and more frequent decontamination of surfaces
Use of face coverings for source control	Face coverings unsuitable for patient use or patient noncompliant with use	Consider modified face coverings, modified methods of securing face coverings, or the use of facility-approved items as face coverings when possible and accepted by the patient
Exposure to cleaning products and disinfectants	Risks associated with patient behaviors (e.g., licking surfaces, attempts to ingest products if accessible)	Have staff members follow instructions on product labels for safe use, including securing products from unauthorized persons such as patients; have staff members dispense individual portions of hand sanitizer directly to patients as needed
Close connections with other high-risk facilities	Regular transfers from facilities at higher risk for SARS-CoV-2 transmission (e.g., homeless shelters, group homes, and correctional facilities)	Develop county and state level plans that support the needs of all higher-risk facilities and address issues such as integrated testing strategies, expanded screening approaches, and community surveillance
**Staff members**		
Physical strain	Time-consuming, frequent wellbeing checks; need for physical restraint of violent/uncooperative patients	Plan for additional or surge workforce capacity; consider flexible leave policies to account for added strain; make provisions for any staff member at higher risk of severe outcomes from COVID-19
Emotional strain	Possible high HCP turnover; potential stigma of working in a psychiatric facility with active SARS-CoV-2 transmission	Plan for additional or surge workforce capacity; develop a communications plan to address stigma
Risk of exposure for clinical care staff members	Patient behavior might increase risk of SARS-CoV-2 exposure (e.g., spitting, licking, thrashing, or intentionally dislodging PPE)	Use modified PPE to allow unrestricted movement and reduce risk of exposure for clinical care staff members working with violent and nonviolent patients (e.g., goggles instead of glasses or face shields, respirators instead of surgical masks, or Tyvek suits instead of gowns)
Risk of exposure for nonclinical care staff members	Security staff members, constantly present on some wards, might be first to respond to a patient issue/violent situation, increasing potential for high-risk exposure; similar risks for transportation staff members who interact with patients during transfer	Use modified PPE to allow unrestricted movement and provide access to utility belts when needed for all nonclinical care staff members (e.g., goggles instead of glasses or face shields, respirators instead of surgical masks, or Tyvek suits instead of gowns)
**Buildings/Wards**		
Social distancing	Open patient wards and rooms to facilitate patient observation; many spaces (including bathrooms) are communal	Control and monitor access to communal areas by symptomatic patients; implement enhanced disinfection practices
Cohorting	Converting single rooms to double occupancy or moving patients to different wards for disease cohorting purposes might be impossible given patients’ different psychiatric needs	Utilize other available structures or facilities when possible
Clinical case management	Units and patient rooms often not set up to provide multifaceted clinical care; for safety reasons, rooms often do not include electric outlets to run medical equipment	Plan for transfer of patients to acute care hospitals as needed

**Abbreviations:** COVID-19 = coronavirus disease 2019; HCP = health care personnel; PPE = personal protective equipment.

## Discussion

SARS-CoV-2 can spread rapidly within congregate residential settings (*2*–*4*), especially complex residential settings such as psychiatric hospitals. Psychiatric facilities often serve several functions concurrently, including long-term care, acute care, detention for psychiatric reasons, memory and addiction treatment, as well as social and behavioral services (*10*). Psychiatric facilities also are often linked to a network of other sites which have an elevated risk of SARS-CoV-2 transmission, including homeless shelters (*3*), group homes, and correctional facilities (*4*). In an outbreak, the interconnectedness of these facilities and the vulnerable populations they serve increase the likelihood of transmission of SARS-CoV-2 between facilities through the admission and discharge of patients and through critical personnel who might work across several facilities.

Following admission of two patients with SARS-CoV-2 infection on April 13, 2020, in the absence of specific guidance on prevention and management of COVID-19 in psychiatric facilities, the state hospital implemented expanded admission screening and IPC procedures. The results of the point prevalence survey, indicating no further transmission among patients and HCP almost 3 weeks after admission of the two SARS-CoV-2-positive patients, suggested that the expanded procedures might have been effective.

Although most health care facilities encounter challenges within an emergency or outbreak context, psychiatric facilities can face unforeseen or compounded issues because of the patient population they serve, their unique workforce, and the constraints of the physical facilities. Psychiatric facilities could possibly reduce the risk of introduction of SARS-CoV-2 by closing or deferring new patient admissions, but these actions would contradict their mandate and result in a backlog of patients at acute care hospitals and other facilities. Therefore, psychiatric facilities need to consider the various IPC, staffing, and structural limitations associated with preventing SARS-CoV-2 transmission in these facilities and plan accordingly. In addition, broader planning at the state and county could be useful in limiting transmission between high-risk facilities, including considerations of an integrated testing strategy, expanded screening protocols, and a community surveillance plan that supports the needs of all high-risk facilities.

The findings in this report are subject to at least four limitations. First, the survey was conducted on one single day; thus, results represent SARS-CoV-2 infection prevalence at a single point in time and recent infections among patients and staff members might not have been detected. Second, SARS-CoV-2 infections might have been missed among the 39% of patients and HCP who did not participate in the survey. Not all patients were willing or able to participate because of their mental or physical states on the day of the survey. In addition, although all HCP were invited to participate, some who were not working on the survey day might not have participated to avoid traveling a long distance to the hospital on a nonwork day. If positive cases were missed among the patients and HCP not tested, true prevalence was higher than indicated by the survey results. Third, answers to survey questions might have been limited by cognitive disabilities or recall bias. Finally, confirmation that the enhanced IPC procedures were responsible for lack of detection of secondary transmission was not possible.

In congregate residential settings, prompt identification of COVID-19 cases and application of strong IPC procedures are critical to ensuring protection of other patients and staff members. Information obtained from this investigation was useful in demonstrating the likely effectiveness of the enhanced, and often resourceful, modified IPC strategies implemented by the state psychiatric hospital. Point prevalence surveys can be useful to monitor outcomes of implementation of IPC measures and to identify cases of COVID-19, including potential asymptomatic cases missed through traditional screening procedures. Successful implementation of this survey suggests that similar surveys would be feasible as an outbreak response activity in this or other psychiatric facilities in the future. For psychiatric facilities in the United States, strong COVID-19 surveillance and response readiness are essential. However, the range of patient behavioral needs makes implementing any universal, uniform measures difficult. Although standard guidance exists for other congregate facilities (*7*) and for HCP in general (*8*), modifications and nonstandard solutions might be required to account for the specific needs of psychiatric facilities, their patients, and staff members. Prevention of transmission in psychiatric facilities will require consideration of the unique risk factors in this population, and approaches might need to be amended to best fit the context of other psychiatric facilities.

**Summary**

**What is already known about this topic?**

SARS-CoV-2 can spread rapidly within residential, congregate settings. Psychiatric facilities are at risk for outbreaks because of patient transfers from other high-risk residential settings and face unique challenges in implementing standard infection prevention and control (IPC) measures because of complex patient needs.

**What is added by this report?**

After admitting two patients with SARS-CoV-2 infection, a psychiatric facility responded by implementing modified and expanded IPC procedures. A point prevalence survey found no evidence of further SARS-CoV-2 transmission within the facility.

**What are the implications for public health practice?**

Adaption of standard IPC strategies in psychiatric facilities to meet patient and facility needs might prevent SARS-CoV-2 transmission, and point prevalence surveys can be useful to assess the likely effectiveness of any adapted IPC measures.

## Footnotes

*
https://www.cdc.gov/coronavirus/2019-ncov/hcp/disposition-hospitalized-patients.html .

† https://www.hhs.gov/ohrp/regulations-and-policy/regulations/45-cfr-46/.
